# ‘Flooding’ using disinformation

**DOI:** 10.1111/bjso.70151

**Published:** 2026-11-08

**Authors:** Ákos Szegőfi, Sacha Altay, Christophe Heintz

**Affiliations:** ^1^ Department of History and Philosophy of Science University of Pittsburgh Pittsburgh Pennsylvania USA; ^2^ Department of Political Science University of Zurich Zurich Switzerland; ^3^ Department of Cognitive Science Central European University Vienna Austria

**Keywords:** disinformation, epistemic vigilance, manipulation, persuasion

## Abstract

Disinformation strategies are often deployed by professionals to elicit confusion rather than to persuade. One key method for this is ‘flooding’: inserting a large number of incongruent propositions into the communication environment, with the goal of preventing people from identifying and acting on reliable information. We assess some of the basic elements of this strategy with two pre‐registered experimental studies (*N* = 2607) on the topics of sunscreens and GMOs. We pitted a flooded versus a non‐flooded information environment against each other, with varying levels of accurate and inaccurate propositions. Our findings suggest that, in some cases, flooding can influence attitudes, push the evaluation of information into the realm of uncertainty and increase subjective feelings of information overload. We further speculate that flooding may elicit rational inattention and have other downstream consequences such as fatigue and passivity.

This constant lying is not aimed at making people believe a lie, but at ensuring that no one believes anything anymore. A people that can no longer distinguish between truth and lies cannot distinguish between right and wrong. (fake Hannah Arendt quote circulating on Twitter/X, Berkowitz, 2024)

## INTRODUCTION

Disinformation, defined as intentionally deceptive content, is sometimes deployed by professionals as a flood: a large amount of material hitting as many communication channels as possible, with the intent to cause confusion or drown out reliable information (Pomerantsev, 2019; Posard et al., 2020). In military studies, this is referred to as ‘censorship through noise’ (Pynnöniemi & Rácz, 2016), or the ‘firehose of falsehood’ model (Paul & Matthews, 2016). Former White House chief strategist Steve Bannon summarized the strategy as: ‘The way to deal with them [the media] is to flood the zone with shit’ (Stelter, 2021). Commentators have suggested that this strategy may be gaining traction in contemporary US politics with President Trump's return to power (for example Klein, 2025).

Famous case studies include the downing of Malaysia Airlines Flight 17 by Russian separatist forces, where flooding was allegedly deployed to confuse audiences about the cause of the catastrophe (Pynnöniemi & Rácz, 2016), climate change disinformation (Fischer et al., 2019; Oreskes & Conway, 2008) and the tobacco industry's efforts from the 1950s to the 1970s to downplay the harms of smoking (Oreskes & Conway, 2010). These cases are sometimes discussed – mainly relying on qualitative methodology – under the topic of *agnotology*, or the study of strategically induced ignorance (Proctor & Schiebinger, 2008).

Although flooding is gaining some attention in the scientific literature (e.g., Anderau, 2023; Rippon, 2024; Steiglechner & Keijzer, 2025), there is no clear evidence on whether the strategy actually works. Psychological mechanisms remain underspecified and untested, as do behavioural outcomes. Most aforementioned accounts treat flooding as a technique aimed at disorienting the target population – an idea echoed in Russian military literature (Chekinov & Bogdanov, 2012; DiResta et al., 2019; Fitzgerald & Brantly, 2017; Giles, 2016; Komov, 1997; Kuleshov, 2014; Lucas & Nimmo, 2015). While military literature does describe flooding in some detail, it cannot be considered a reliable basis to decide whether flooding is dangerous or not. One pervasive problem is that professionals behind influence operations have vested interests in overstating their success rates. Cambridge Analytica, for example, made largely unfalsifiable claims of having swung the 2016 US election as well as the Brexit referendum to advertise its services (Farina et al., 2025). Available evidence on the effectiveness of Russian election interference in Africa is similarly dubious (Shekovtsov, 2023). Practitioners have reason to make themselves look more dangerous than they are, and taking their claims at face value can actually contribute to the success of these operations by spreading confusion and undermining trust in democratic processes (Altay & Acerbi, 2023; Huang & Cruz, 2022; Nisbet et al., 2021).

Assessing the effectiveness of influence operations is a challenge that goes well beyond defence research. The testimonies of famous Soviet defectors (Bittman, 1972; Shultz & Godson, 1984; Pacepa & Rychlak, 2013) describe disinformation operations as traditionally long‐term affairs, with operators expecting ‘cumulative effects’ over time (Kux, 1985). Curiously, one of the most referenced sources of such operations, defector Vasilii Mitrokhin, reported never having seen any analysis on operation success rates – despite being an archivist at the KGB (Andrew, 2000). There is a suggestion that disinformation operations rely on fairly standard metrics, for example the penetration of narratives across the media landscape: how many outlets pick up a narrative, and how many social media platforms it reaches. For example, Hoyle and Slerka (2024) describe operators as tracking public opinion polls, policy changes plausibly linked to disinformation, and notable public behaviours (protests, strikes, and similar) that may have been triggered by influence operations. These approaches typically focus on whether a manipulative, false narrative gets covered by reputable outlets, as duping a credible source into circulating disinformation is taken as one of the main signs of success (Ong & Cabanes, 2018). A ‘Breakout Scale’ has also been proposed to assess the danger posed by influence operations, based on social media penetration, uptake by mainstream media and celebrities, and policy changes (Nimmo, 2020). Unfortunately, none of these methods allow us to draw causal inferences about disinformation changing beliefs or attitudes (let alone behaviours). It is well‐known that deceptive narratives can spread without audiences believing them (Wong & Burkell, 2017), and when public opinion does shift, the actual causes may lie elsewhere (political events, socio‐economic factors).

If, as case studies suggest, the strategy is indeed effective, then we must understand why and how it works. In this paper, we experimentally test the causal effect of the flooding strategy on beliefs and attitudes and speculate about potential behavioural and long‐term effects. We begin by detailing a cognitive‐psychological model of flooding that allows us to draw testable predictions. We then present our experimental approach and results. We close with a general discussion on what our results tell us about the proposed model, the limitations and outline some potential future research directions.

## THE PSYCHOLOGY OF FLOODING

We characterize flooding as a deceptive communication strategy that consists in increasing the number of propositions that are inconsistent with the flooded (factual) proposition – and often inconsistent with each other. In this sense, flooding differs from the repetition‐based mechanisms central to the misinformation and propaganda literature (for a review of the repetition‐induced truth effect, see Unkelbach & Koch, 2019). The aim of flooding is presumably to prevent audiences from believing that a (reliable) target proposition is true. When the target proposition is flooded with alternative, false propositions, the goal is not necessarily to provide specific ‘debunks’ or direct contradictions to the target claim – as there might not be any strong counter‐arguments available – rather, to expose the audience to a large amount of heterogeneous, conflicting information on a broader topic. Therefore, the general impression that the audience may form is something like ‘there appears to be considerable disagreement and confusion around this topic.’ In our account, this should have somewhat asymmetric effects on the evaluation of reliable, accurate claims and on unreliable, inaccurate ones. When an otherwise credible proposition appears within a communication environment containing multiple conflicting claims, it risks becoming ‘just another statement’ among many other claims, in an apparently unsettled – and increasingly tedious‐looking – debate. Due to this, flooding would primarily reduce confidence in reliable, accurate information rather than systematically increase confidence in any particular inaccurate proposition.

The mechanism we hypothesize behind this effect is that, with the flood of contradictory information, audiences become uncertain, as they perceive overall confusion and/or debate around the topic in question. Audiences then make an estimate of the expected cognitive costs of reducing this uncertainty – for example, the costs of ‘figuring out’ what is going on – and if this estimate of costs appears to be larger than the expected cognitive benefits of acquiring a good enough understanding of what is going on, then audiences stop updating their beliefs on the given topic, and may disengage from it altogether (e.g., stop seeking out information about the topic in the future). Our proposed cognitive mechanism rests on the cognitive model of epistemic vigilance: the human psychological capacities that assess the veracity of communicated information (see Mercier, 2020; Sperber et al., 2010; Watson & Morgan, 2025). The predictions we draw about how flooding should impact the human mind are consistent with how epistemic vigilance is hypothesized to function. When the capacities of epistemic vigilance are overloaded – as there is too much communicated content available – the result is that audiences simply stop listening to what is said. In this sense, epistemic vigilance reads the contradictions and the volume of heterogeneous information as a form of evidence that no clear answer can be reached on a topic. The mechanism described here is important as it is in contrast with the social psychological (and some psycholinguistic) literature, which is otherwise in agreement with the idea that humans do indeed check the veracity of communicated information. The presumption there would be is that if cognitive capacities – for any reason – are exhausted, then the result is credulous belief in whatever is communicated (e.g. Gilbert et al., 1990; Gilbert & Malone, 1995; also see truth‐default theories like that of Levine, 2014; and dual‐processing theories of misinformation, like Bago et al., 2020). In this debate, we are inclined to side more with the epistemic vigilance framework. It seems unlikely to us that in a situation involving a lot of cacophony, where we do not quite understand what is going on and begin to feel overloaded and tired, our baseline psychological reaction is to start unselectively believing everything we are told. From an adaptive standpoint, this prediction does not make too much sense. What seems more likely is that instead of believing, we put our hands on our ears and stop listening, until the deluge of communicated content is mediated to a level that our cognitive apparatus can track. While this cognitive feature of ‘closed doors policy’ (e.g., no belief update if capacities of epistemic vigilance are not engaged) might be adaptive in most situations involving communication, there can be disadvantages. Every now and then, valuable and credible information and sources can be found inside the cacophony, which should be used for belief‐updating, but become discounted alongside the rest of the noise. It would seem that the influence operations built on flooding have an intuitive grasp of this adaptive, but exploitable feature of the mind.

In our confusion‐focused model depicted in Figure 1, flooding the communication environment with incongruent propositions first produces a sense of uncertainty in the audience, as well as a subjective feeling of information overload – a general impression that it is difficult to figure out what is going on about a specific topic due to the abundance of information (Roetzel, 2018; Eppler & Mengis, 2004). With more content added to the communication environment, the cost of information processing grows, while expected cognitive benefits of knowing the ‘state of things’ drops. Given that the costs of reducing this uncertainty are high enough, this process leads to rational inattention, where audiences no longer engage with information on a specific topic, as they estimate that their limited cognitive capacities and time are best spent elsewhere (Sims, 2006; Maćkowiak et al., 2021.) We would risk speculating that rational inattention has possible downstream consequences, such as changes in information search patterns, as well as inaction/passivity of the audience (provided that reliable information cannot be recovered, it becomes impossible to act on it.) We find this latter notion of ‘inaction’ important, for the reason that the more persuasion‐based approaches to disinformation often involve a different prediction: namely, that the outcome – and ultimate goal – of spreading disinformation is the creation of radicalized, ultra‐active individuals (e.g. Akram & Nasar, 2023; Piazza, 2022; Benkler et al., 2018). To summarize our reading: flooding aims to leave listeners confused, overloaded and ultimately uninterested in the topic that was flooded. It erodes confidence in reliable messages, makes the identification of trustworthy sources cumbersome, and finally, it may also impair one's ability to efficiently navigate the respective communication environment. In the discussion section, we further speculate about how flooding may alter the perception of the respective communication environment as well, calling into question whether the truth is even retrievable from the existing network (e.g., cultivating opinions like ‘Facebook is a mess’, downstream from ‘the debate around this topic is a mess’).

**FIGURE 1:**
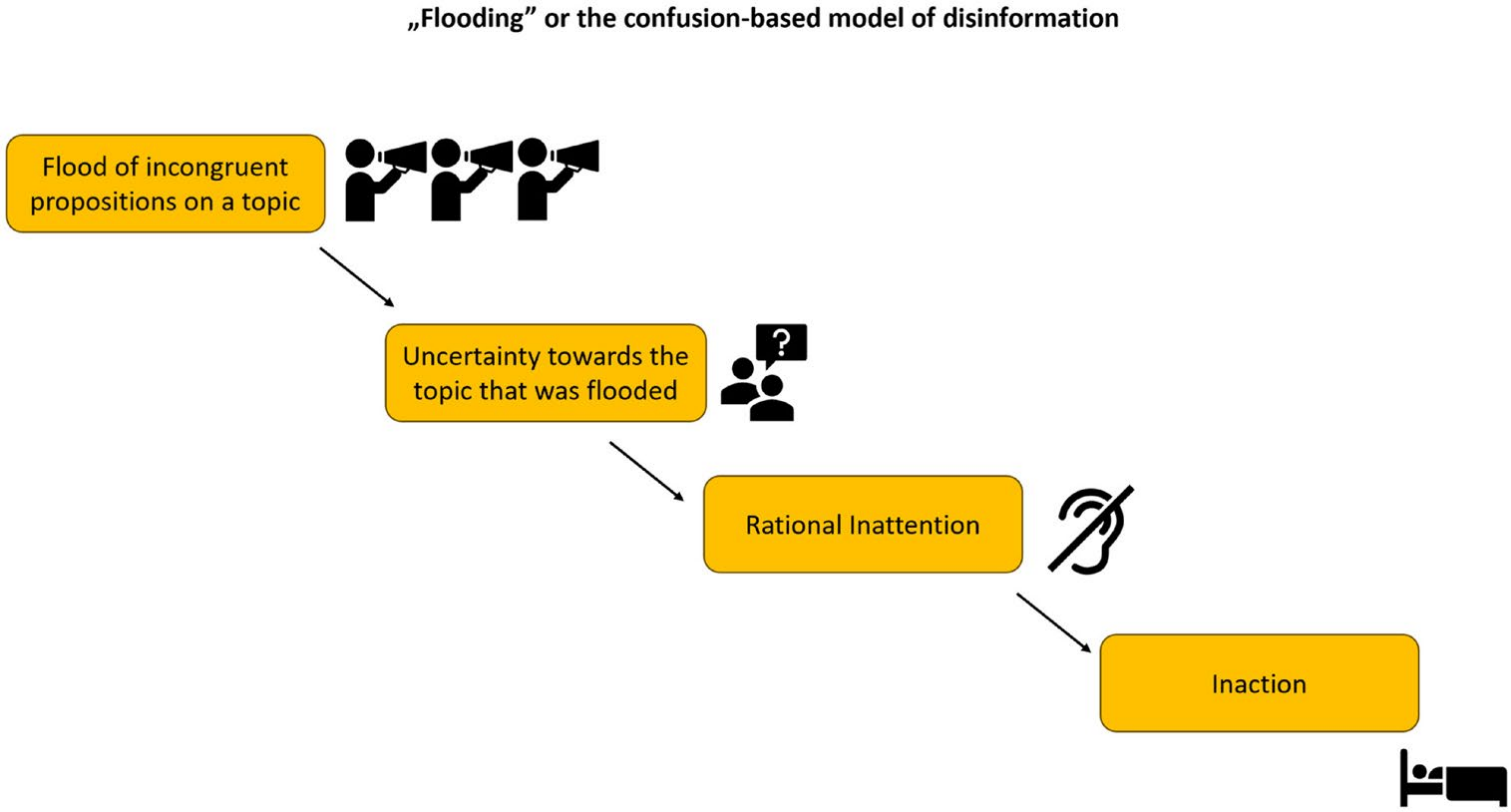
From flooding to supposed behavioural outcome.

## IMPLICATIONS FOR PRESENT STUDY

In our two experiments, we have restricted ourselves to testing the most basic elements of our model: uncertainty and information overload. Some of the predictions mentioned in the previous section – like rational inattention and inaction – were beyond the scope of our designs. We put in place another restriction, which had to do with sources. In both studies, accurate target propositions in all cases were communicated by sources endowed with cues of relevant expertise, while inaccurate flooding propositions were always shared by sources with irrelevant expertise. While we are aware that influence operations ‘in the wild’ would absolutely try to make their sources look as if they were experts, we reasoned that flooding is primarily a content‐related manipulation; therefore, we first wanted to register purely content‐related effects, which called for keeping source‐related effects constant. To achieve this, we have designed an ‘ideal’ communication environment, where truth was always told by experts, and flooding messages were always communicated by random people, and these cues were easily retrievable. We reasoned that if we are able to register flooding effects even in this minimal environment, then any other – source‐related – manipulation should only increase its strength.

We therefore primarily focused on checking if flooding indeed elicits uncertainty from audiences in a way that decreases confidence in actually accurate messages, and whether it cultivates a feeling of information overload. Apart from measuring the perceived accuracy of standalone messages, we also measured generic attitudes towards the topic that was flooded. We did this both pre and post manipulation to acquire an overarching idea on how our participants change – or don't – their perception of the experimental topic. Therefore, in this current form, the two experiments reported in this paper do not test the entire framework put forward in the previous section. Nonetheless, we found it important to show the whole ‘blueprint’ of our model so that other studies may continue building on the fundamentals we laid down. The minimal predictions tested in our experiments were the following:

1. The attitude scores of participants who are exposed to the flooded condition would be significantly lower compared to the attitude scores of participants in the Control condition.
2. The accuracy scores for the accurate propositions in the flooded condition will be significantly lower than the accuracy scores for accurate propositions in the Control condition.
3. There would be no significant difference in the accuracy scores of the inaccurate flooding propositions between the two conditions.
4. The information overload score of the participants exposed to the flooding condition would be significantly higher compared to the information overload score of participants exposed to the Control condition.

Please note that in the pre‐registration, predictions 2 and 3 were put down as one prediction. We thought that for clarity, it is better to present them here as two separate predictions. Given that significant effects are found, we are to conduct additional exploratory analyses. These involved calculating a truth‐discernment metric (average ratings of accurate propositions minus average ratings of inaccurate propositions) with the expectation that the difference was smaller in the flooded condition. Another is using a regression that would look at perceived accuracy as a function of pre‐treatment attitudes, with the expectation that flooding influences the perceived accuracy of statements *even* if participants have strongly supportive views towards the topic that was flooded.

### Procedure

To test our predictions, we designed two online, vignette experiments (anonymized pre‐registration https://osf.io/aebxz/overview?view_only=db192803c86e4043911ad733bb7f4de1) using two debated, but not explicitly political topics: sunscreen safety and GMOs. Participants were recruited on Prolific Academic and, after agreeing to participate, were re‐directed to a mock social media feed implemented on the survey platform Qualtrics XM. We used several built‐in features to ensure data quality, such as bot detection and preventing multiple submissions from the same user. The individual posts of the feed were generated using a free‐to‐use online ‘Tweet Generator’ that mimicked the appearance of social media posts from X/Twitter. The content of posts was written specifically for the experiments, but we drew inspiration from common misconceptions on sunscreen usage and GMO safety. Five accurate target propositions and five inaccurate flooding propositions were used per experiment. In the control condition, one accurate and one inaccurate proposition were pseudo‐randomly sampled for each participant, making it a communication environment with a 1:1 truth‐to‐false ratio. In the experimental condition, one accurate target proposition was pseudo‐randomly sampled from the set of five accurate propositions, while all five inaccurate flooding propositions were presented to participants in a random order, making this environment the flooded, 1:5 truth‐to‐false ratio.^1^ We settled on this counter‐balancing method to avoid the effects of one inaccurate proposition accidentally having a direct debunking effect on an accurate proposition. There were no anonymous sources in our experiment; all sources had surnames and their profession/workplace in their avatars. To control for any gender or racial bias, we used initials instead of full first names and decided not to add any profile pictures. Examples of how posts were displayed in the experiment are shown in Figure 2.

**FIGURE 2:**
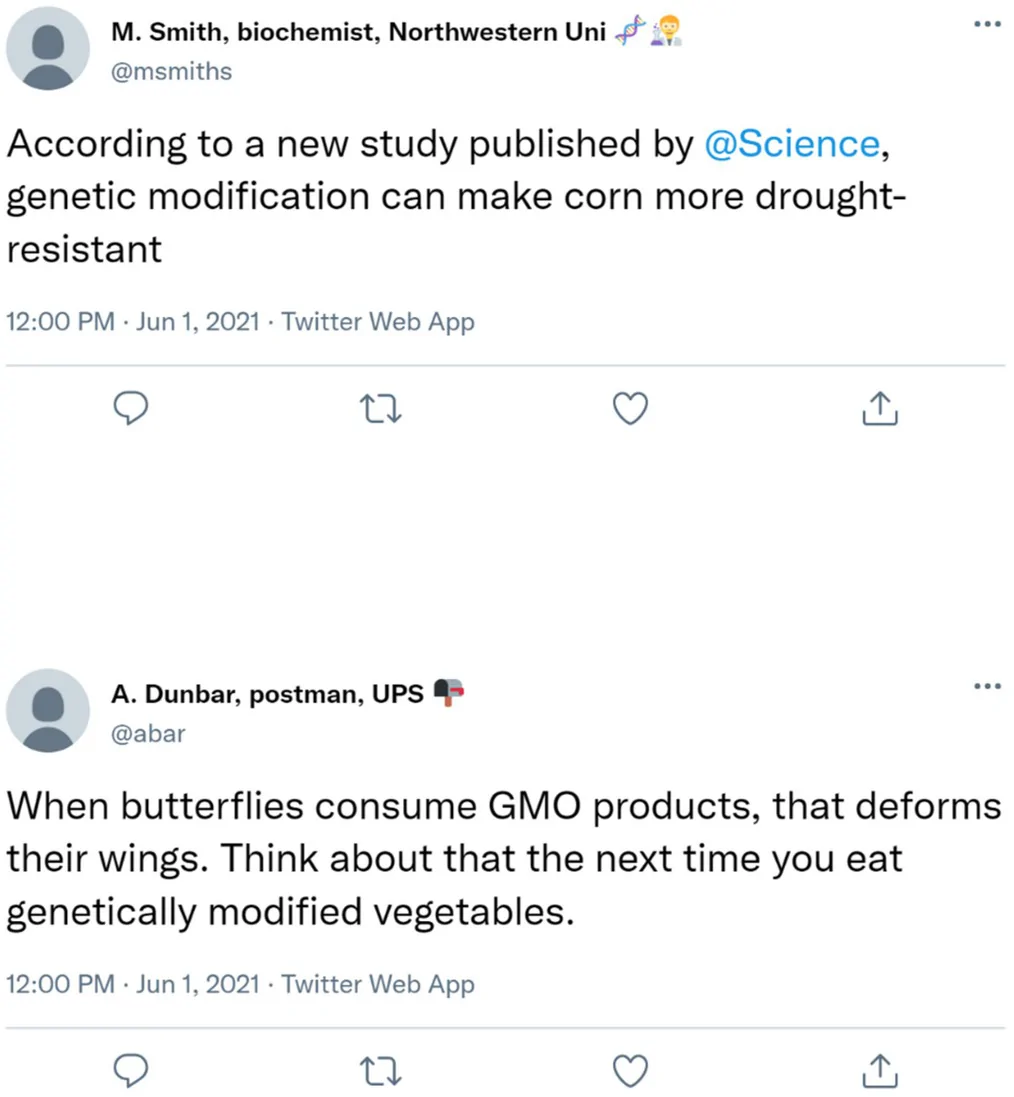
Appearance of posts – one accurate (top) and one inaccurate (bottom) claim – on the simulated newsfeed.

After agreeing to the consent form, participants were randomly allocated to either Study 1 (sunscreens) or Study 2 (GMOs). Within each experiment, they were again randomly allocated to either the control or the experimental condition. Before seeing the newsfeed, participants answered a pre‐treatment survey question about general attitudes towards sunscreens/GMOs. The stimulated newsfeed was followed by the perceived accuracy, the information overload and finally, the post‐attitude measure. Participants then saw a comprehensive debriefing on the actual accuracy of the stimuli they saw in the study with references to regulatory bodies and scientific evidence. They were finally redirected to Prolific Academic with a completion code.

### Sample

Participants were recruited through Prolific Academic. We tested a US sample, with English as a first language as a customized filter, to ensure that our participants perfectly understood the instructions and the experimental stimuli. We conducted an a priori power analysis in G*Power (Faul et al., 2007), which indicated a sample of 1302 participants per experiment for a two‐tailed test of difference between two independent means at *d* = 0.2 (alpha = 5%, power = 95%). Given that we ran two experiments simultaneously, our total target sample size was 2604 participants (one participant could only participate in one experiment). We ultimately recruited *N* = 2607. Study 1, focusing on sunscreens, had a sample size of *n* = 1307, while Study 2, focusing on GMOs, had *n* = 1300, where the sensitivity power analyses showed 95% power for both studies to detect a minimum of *d* = 0.2 (two‐tailed). All data analyses were conducted in R (R version 4.4.1, http://www.r‐project.org).

### Dependent variables and analysis

To register generic changes in how participants see the experimental topics in general, we measured pre‐ and post‐treatment attitudes with regard to sunscreen/GMO safety on a 7‐point Likert scale, from ‘I disagree completely’ to ‘I agree completely’, with an ‘I don't know’ answer corresponding to the mid‐value of 4, using an ‘agreement with statement’‐type measure. In the case of sunscreens, the statement was: ‘Sunscreen is safe and crucial for avoiding sunburn and minimizing the risk of skin cancer.’ For GMOs, on the other hand, we captured two different types of attitudes. The first was on the environmental impact of cultivating GMOs, with the statement: ‘GMOs are safe for the environment.’ And another one on consuming GMO products, with the statement: ‘GMOs are safe to consume.’ In both experiments, the attitude measures were repeated post‐treatment.

To measure the effect of flooding on individual messages, we relied on a standard perceived accuracy question. Following the experimental manipulation, each participant rated two propositions – the one target and one flooding‐proposition – on a 7‐point Likert scale, from ‘completely inaccurate’ to ‘completely accurate,’ with an ‘I don't know’ answer corresponding to the mid‐value of 4. The descriptives on stimuli accuracy can be seen in Table 1. Given that in the control condition, there was one accurate and one inaccurate statement (randomly sampled from the set of total propositions), we simply asked participants about their accuracy. However, given that in the experimental condition there was one accurate (randomly sampled) and five inaccurate (in random order) statements, we posed the accuracy question about the one accurate statement, and then one pseudo‐randomly sampled inaccurate statement. The target propositions were invariably accurate according to current scientific understanding, while the flooding (inaccurate) propositions were written to be inaccurate according to current scientific understanding, but not unbelievable.

**TABLE 1:** Stimuli descriptives.

Stimuli	*M*	SD	Median	Skew	Kurtosis
Sunscreen accurate items/control	5.22	1.60	6	−0.95	0.16
Sunscreen accurate items/flooding	5.01	1.67	5	−0.79	−0.15
Sunscreen inaccurate items/control	3.70	1.54	4	−0.05	−0.66
Sunscreen inaccurate items/flooding	3.94	1.56	4	−0.16	−0.58
GMO accurate items/control	4.71	1.46	5	−0.58	−0.12
GMO accurate items/treatment	4.55	1.48	5	−0.56	−0.14
GMO inaccurate items/control	4.09	1.68	4	−0.19	−0.83
GMO inaccurate items/treatment	4.21	1.70	4	−0.22	−0.87

We then measured information overload with the following statement: ‘I find it difficult to figure out what's going on with sunscreens/GMOs’, on a 7‐point Likert scale (from ‘I disagree completely’ to ‘I agree completely’ with an ‘I'm not sure’ answer corresponding to the mid‐value of 4). Statistical analyses for each prediction were calculated using two‐tailed OLS linear regression models, with Age and Gender as covariates.

## RESULTS

### Study 1

A total of 1307 participants (Female = 722; *M*_age_ = 41.8, SD = 12.91) completed the sunscreen‐themed experiment. For a visualization of all the main test results, please see Figure 3. First, we estimated the effect of flooding on attitudes – while controlling for attitudes before the treatment. Participants' pre‐treatment attitudes towards sunscreens were generally positive (*M* = 5.76, SD = 1.28). Contrary to our expectations, participants in the flooding condition reported more positive post‐treatment attitudes towards sunscreen compared to the control group, *b* = 0.138, *t*(1301) = 3.281, 95% CI [0.056, 0.221], *p* = .001.

**FIGURE 3:**
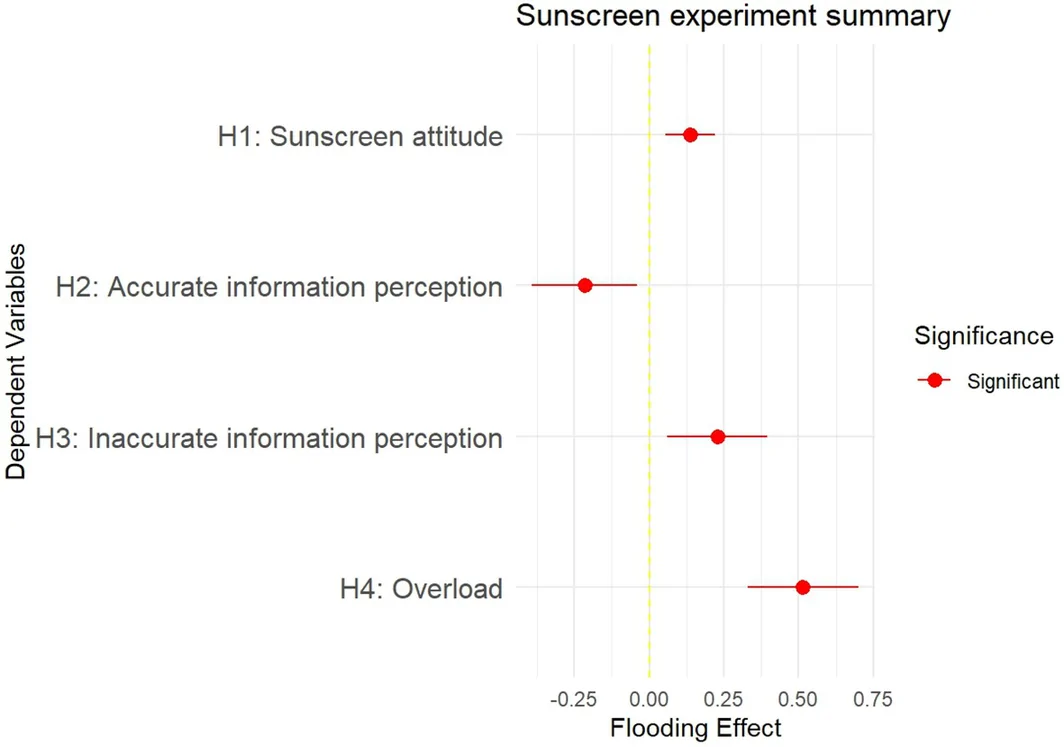
Results of Study 1.

Second, we tested the effect of flooding on the perceived accuracy of target and flooding propositions. As expected, the treatment significantly reduced the perceived accuracy of accurate target propositions, *b* = −0.216, *t*(1302) = −2.404, 95% CI [−0.393, −0.040], *p* = .016. On average, participants rated these items as fairly accurate (*M* = 5.12, SD = 1.64). Unexpectedly, however, the flooding condition also led to significantly higher accuracy ratings of inaccurate propositions, *b* = 0.228, *t*(1302) = 2.670, 95% CI [0.061, 0.396], *p* = .008. Ratings of these inaccurate items fell on the more inaccurate end of the scale (*M* = 3.82, SD = 1.56). To elaborate more on this finding, an exploratory analysis was run specifically on truth‐discernment using a linear mixed‐effects model with random intercepts for participants and stimuli items (see Figure 4). Perceived accuracy was modelled as the dependent variable, with the interaction between a compound variable called ‘Discernment’ (accurate vs. inaccurate) and ‘Condition’ (control vs. treatment) as the fixed effect. The Discernment variable was computed by aggregating all accurate ratings and all inaccurate ratings, then taking their difference. We found that participants in the flooded environment showed worse truth‐discernment than participants in the control (*b* = −0.45, *t*(2525.85) = −3.62, 95% CI [−0.69, −0.20], *p* < .001). Taken together with the basic finding, flooding seems to push the evaluation of all information towards ‘I don't know’ answers (the midpoint of 4 on the Likert scale).

**FIGURE 4:**
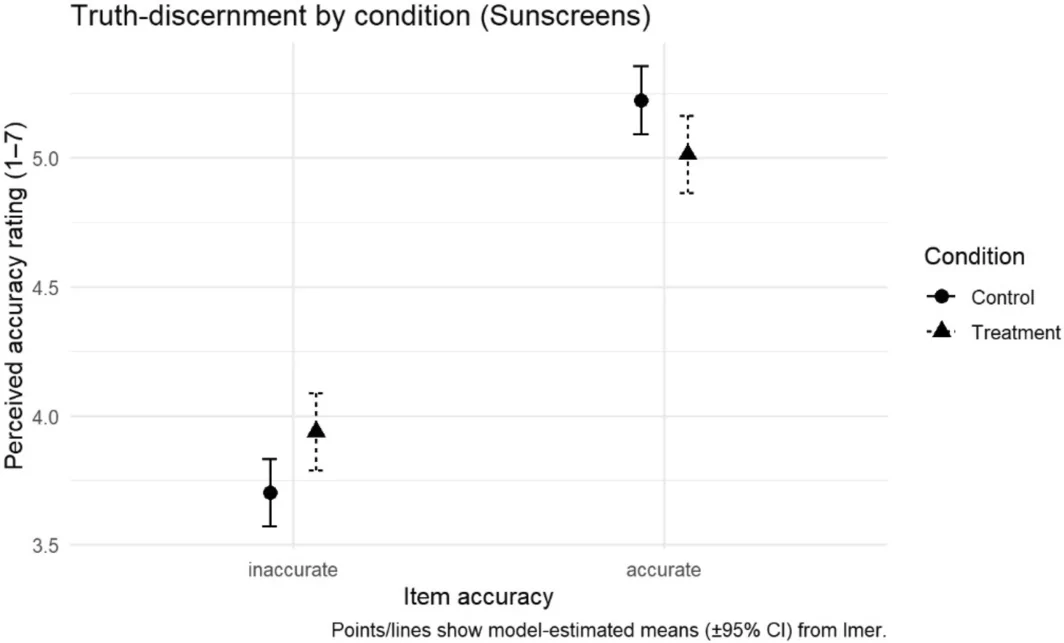
Exploratory analysis on truth‐discernment in Study 1. For additional details, see Table A9 in the Supplementary Materials.

Third, in accordance with our prediction, participants in the flooding condition reported significantly higher feelings of information overload (*M* = 4.02, SD = 1.75), *b* = 0.515, *t*(1302) = 5.427, 95% CI [0.329, 0.701], *p* < .001. For main analysis results of Study 1, please see Table 2.

**TABLE 2:** Summary of Study I results including point estimates (unstandardized regression coefficients), confidence intervals and *p*‐values. For full regression tables with SD and *t*‐values, model statistics, age and gender controls, see Supplementary Materials.

Dependent variable	Predictor	*b*	95% CI	*p*
Post‐treatment sunscreen attitude	Intercept	2.177	[1.928, 2.426]	–
	Pre‐treatment attitude	0.680***	[0.648, 0.713]	<.001
	Condition (treatment)	0.138**	[0.056, 0.221]	.001
Accurate information perception	Intercept	5.696	[5.369, 6.024]	–
	Condition (treatment)	−0.216*	[−0.393, −0.040]	.016
Inaccurate information perception	Intercept	3.949	[3.638, 4.260]	–
	Condition (treatment)	0.228**	[0.061, 0.396]	.008
Information overload	Intercept	3.932	[3.587, 4.278]	–
	Condition (treatment)	0.515***	[0.329, 0.701]	<.001

*p < .05, **p < .01, ***p < .001.

### Study 2

A total of 1300 participants (Female = 721; *M*_Age_ = 41.51, SD = 12.74) completed the GMO‐themed experiment. The main test results are visualized in Figure 5. First, we estimated the effect of flooding on attitudes towards GMOs – while controlling for attitudes before the treatment. Participants' pre‐treatment attitudes towards GMOs were generally agnostic (*M* = 4.33, SD = 1.69 for GMO attitude 1 and *M* = 4.11, SD = 1.57 for GMO attitude 2). In line with our prediction, compared to the Control group, participants in the flooding condition reported more negative attitudes towards GMO consumption (GMO attitude 1), *b* = −0.167, *t*(1294) = −3.899, 95% CI [−0.250, −0.083], *p* < .001 and more negative attitudes towards the environmental impact of cultivating GMOs (GMO attitude 2), *b* = −0.192, *t*(1294) = −4.326, 95% CI [−0.280, −0.105], *p* < .001.

**FIGURE 5:**
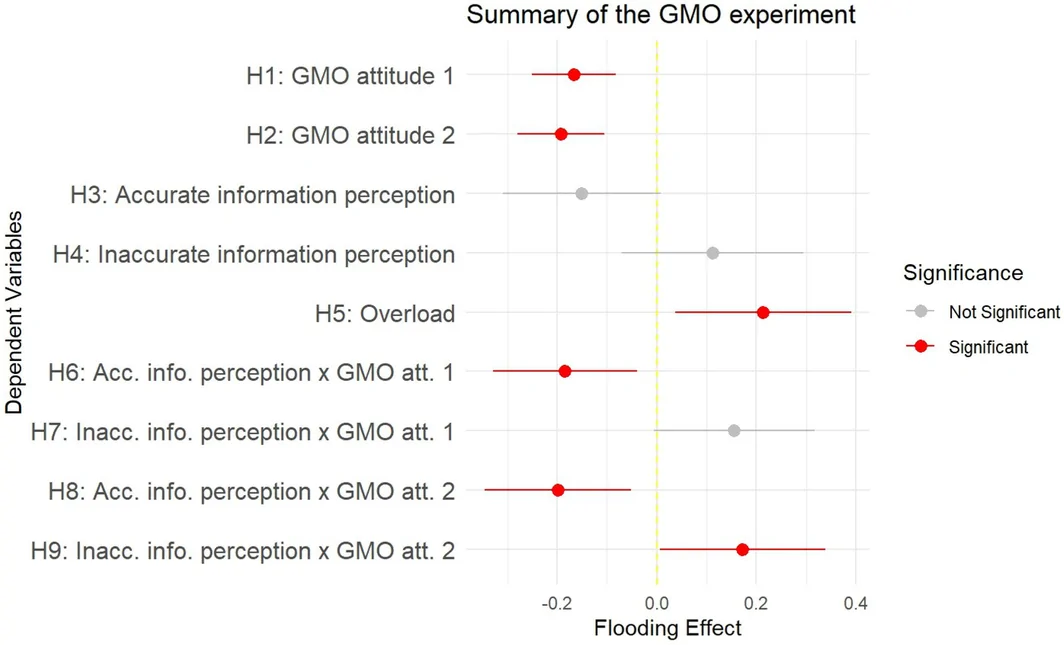
Visualization of results from Study 2, including the exploratory analysis on the interaction between GMO attitudes 1, 2 and the perceived accuracy ratings in the last four rows.

Second, we tested the effect of flooding on the perceived accuracy of accurate and inaccurate propositions about GMOs. Participants in the Flooding condition gave lower ratings to accurate posts, but this effect, while in the expected direction, was not statistically significant, *b* = −0.151, *t*(1295) = −1.867, 95% CI [−0.309, 0.008], *p* = .062. As predicted, participants in the Flooding condition did not change their perceived accuracy ratings of inaccurate flooding propositions, *b* = 0.112, *t*(1295) = 1.206, 95% CI [−0.070, 0.295], *p* = .228.

We also tested moderation effects and found that participants with more positive pre‐treatment attitudes towards consuming GMOs (GMO Attitude 1) were significantly more likely to recognize accurate posts as accurate, *b* = 0.365, *t*(1294) = 16.348, 95% CI [0.321, 0.409], *p* < .001. But this ability was significantly undermined by flooding, *b* = −0.184, *t*(1294) = −2.501, 95% CI [−0.329, −0.040], *p* = .012. In other words, even among those with strong positive attitudes towards GMO consumption, exposure to high volumes of inaccurate content reduced their capacity to identify accurate information. When it comes to inaccurate posts, participants with more positive attitudes were significantly less likely to rate them as accurate, *b* = −0.477, *t*(1294) = −19.128, 95% CI [−0.526, −0.428], *p* < .001. However, unlike for accurate items, the flooding manipulation did not significantly alter this relationship, *b* = 0.156, *t*(1294) = 1.894, 95% CI [−0.006, 0.317], *p* = .058. The same exploratory analysis was run on attitudes towards the environmental impact of GMOs (GMO Attitude 2). These results mirrored our earlier findings: participants with more positive attitudes rated accurate information as more accurate, *b* = 0.358, *t*(1294) = 14.621, 95% CI [0.310, 0.406], *p* < .001 and flooding significantly weakened this relationship, *b* = −0.199, 95%, *t*(1294) = −2.656, CI [−0.346, −0.052], *p* = .008. Similarly, there was a strong negative relationship between positive attitudes and perceived accuracy of inaccurate posts, *b* = −0.448, *t*(1294) = −16.137, 95% CI [−0.502, −0.393], *p* < .001, along with a marginal effect of the flooding, *b* = 0.172, *t*(1294) = 2.030, 95% CI [0.006, 0.339], *p* = .043.

Lastly, as predicted, the flooding condition significantly increased perceptions of information overload, *b* = 0.214, *t*(1295) = 2.374, 95% CI [0.037, 0.391], *p* = .018. The main statistical results for Study 2 can be found in Table 3.

**TABLE 3:** Summary of Study II results including point estimates (unstandardized regression coefficients), confidence intervals and *p*‐values. For full regression tables with SD, *t*‐values, model statistics, age and gender controls, see the Supplementary Materials.

Dependent variable	Predictor	*b*	95% CI	*p*
GMO Attitude 1	Intercept	0.424	[0.233, 0.615]	–
	Pre‐treatment attitude	0.895***	[0.869, 0.920]	<.001
	Condition (treatment)	−0.167***	[−0.250, −0.083]	<.001
GMO Attitude 2	Intercept	0.567	[0.371, 0.764]	–
	Pre‐treatment attitude	0.864***	[0.835, 0.892]	<.001
	Condition (treatment)	−0.192***	[−0.280, −0.105]	<.001
Accurate information perception	Intercept	4.791	[4.496, 5.085]	–
	Condition (treatment)	−0.151	[−0.309, 0.008]	.062
Inaccurate information perception	Intercept	4.277	[3.938, 4.616]	–
	Condition (treatment)	0.112	[−0.070, 0.295]	.228
Information overload	Intercept	4.697	[4.369, 5.026]	–
	Condition (treatment)	0.214*	[0.037, 0.391]	.018
Accurate information (× GMO Attitude 1)	Intercept	3.199	[2.870, 3.528]	–
	Pre‐treatment attitude	0.365***	[0.321, 0.409]	<.001
	Condition (treatment)	−0.184*	[−0.329, −0.040]	.012
Inaccurate information (× GMO Attitude 1)	Intercept	6.355	[5.988, 6.723]	–
	Pre‐treatment attitude	−0.477***	[−0.526, −0.428]	<.001
	Condition (treatment)	0.156	[−0.006, 0.317]	.058
Accurate information (× GMO Attitude 2)	Intercept	3.387	[3.055, 3.719]	–
	Pre‐treatment attitude	0.358***	[0.310, 0.406]	<.001
	Condition (treatment)	−0.199**	[−0.346, −0.052]	.008
Inaccurate information (× GMO Attitude 2)	Intercept	6.033	[5.657, 6.409]	–
	Pre‐treatment attitude	−0.448***	[−0.502, −0.393]	<.001
	Condition (treatment)	0.172*	[0.006, 0.339]	.043

*p < .05, **p < .01, ***p < .001.

Taken together with Study 1, the GMO findings provide a more complete picture. While participants with strong prior attitudes were generally better at distinguishing accurate from inaccurate information, flooding can impair this ability – especially when it comes to evaluating accurate content. This would then contribute to confusion and information overload.

One might notice that the exploratory analyses conducted for the two experiments were different. This is because in Study 1, we reported significant effects of flooding decreasing the perceived accuracy of true propositions; therefore, an exploratory study on truth‐discernment was conducted. Meanwhile, Study 2 showed consistent and strong effects on (both) types of attitudes included in the design; therefore, we decided to conduct an exploratory analysis focusing on these. Provided that we believe conducting exploratory analyses on non‐significant results constitutes bad practice (e.g., playing with different comparisons until something shows up significant), we decided to omit these tests.

## GENERAL DISCUSSION

The studies included in this paper tested a model in which disinformation works by generating uncertainty and cultivating a subjective feeling of information overload. Our findings offer suggestive evidence that flooding may produce such effects. We tested three predictions emerging from our cognitive model of flooding.

First, flooding should reduce the perceived accuracy of true propositions while leaving inaccurate propositions roughly unchanged. Study 1 supported this only in part: flooding importantly reduced ratings of accurate posts but, unexpectedly, also raised ratings of inaccurate ones. In practice, this meant that both the ratings of accurate and inaccurate information began converging towards ‘I don't know’ answers in the experimental condition. An exploratory analysis revealed that truth‐discernment between accurate/inaccurate information dropped overall, which is consistent with the idea that flooding pushes evaluations towards ‘I don't know’ answers rather than towards strong beliefs in falsehoods. The result on accuracy ratings in Study 2 supported the idea that flooding does not meaningfully increase the perceived accuracy of any individual false statements; however, it also failed to register any direct detrimental effect on individual pieces of reliable information. One possible interpretation is that, in Study 1, participants became less willing to make strong accuracy judgements in either direction, as most people would agree with the fact that sunscreens are necessary. Study 2 did not reproduce this symmetrical pattern; however, we consider the attitude‐related findings as evidence that flooding indeed shifted perceptions on the topic, and in the expected direction.

This leads us to our attitude‐related findings. According to our predictions, flooding should shift general attitudes on the target topic – that is, have an impact on the overall perception of ‘sunscreens’ or ‘GMOs.’ While we found the converse effect on sunscreens, both GMO‐related attitudes decreased due to flooding in the expected direction. Encouraged by the results in Study 2, we then conducted an exploratory analysis where we looked at prior attitudes in interaction with perceived accuracy ratings. The test revealed that even among participants who viewed GMOs initially favourably, flooding undermined the recognition of accurate posts compared to the control. Taken together with the findings from Study 1, we would conclude that flooding indeed increases uncertainty and that this effect may even be observable among those with confident priors.

Third: flooding should increase subjective feelings of information overload. Both studies confirmed this. While it may sound obvious that flooding someone with loads of incongruent information is going to produce such an effect, it was still important to register. The feeling of information overload is treated as a predecessor to other phenomena presumed by our model: most importantly, rational inattention (on the relation of the two, see: Van Zandt, 2004; Persson, 2018), by which our model predicts disengagement and ultimately inaction.

It must be reiterated that our results looked at short‐term attitudes and beliefs, and studies were not designed to test the entire cognitive model outlined in the introduction. For example, the behavioural prediction that flooding causes disengagement and inaction possibly requires a longitudinal strategy and different operationalization. Rational inattention would most probably require measures having to do with changes in information search patterns (with eye‐tracking or dwelling time metrics), or some implicit approach of capturing the willingness of audiences to keep learning more on‐topic information.

Several other limitations signposted in antecedent sections of this paper should temper our interpretation. The two experiments relied on keeping source‐related cues constant across conditions. This resulted in an ‘idealized’ communication environment, where accurate posts always came from sources with relevant expertise (dermatologists, M.D.s) and inaccurate posts always came from sources with irrelevant expertise (marketing specialists, lifestyle coaches). Real‐life flooding operations actively try to ‘hack’ their sources to make them appear competent (Donovan & Freidberg, 2019). Holding expertise cues constant in our design probably underestimates the field effect. Another limitation deserving a note is the truth‐to‐falsehood ratio. We contrasted 1:1 and 1:5 conditions. We intentionally settled on this large gap to detect basic effects; future work should test intermediate ratios and how intensity varies over time, provided that real flooding operations are not constant. More detailed tests could also reveal diminishing returns of adding more and more information on a topic. Finally, the topics featured in our studies (sunscreens and GMOs) are not explicitly partisan. Politically more charged topics, where flooding is most often deployed (Oreskes & Conway, 2008; Wilder, 2021), may produce different dynamics.

In addition to the question of politically polarizing content, one might ask to what extent flooding effects would vary depending on the topic that is being flooded.^2^ Such a question is warranted given that meaningful variation was already observable between our studies: GMO‐stimuli appeared to work somewhat differently on participants compared to sunscreen ones. What explains this? Here we would speculate that flooding effects may be stronger when already contested topics are targeted. That is: it should be less effortful to make an existing debate appear a lot worse than to create the illusion of a non‐existing, tedious debate from scratch. From this perspective, the conventional wisdom about influence operations, namely that they tend to target already existing societal ‘vulnerabilities’ – for example, sensitive, polarizing issues in a target society – makes sense. GMO safety, for example, at least in a US context, is arguably a more polarizing issue than sunscreen usage. Another point we could offer here is that influence operations aiming to flood a novel topic must overcome a notable psychological problem having to do with the role of information ‘surprisingness’ in belief updating (e.g., Filipowicz et al., 2018). Any new information that, in one way or another, goes against topics that audiences believe to be well‐established and/or enjoy some consensus will probably spark interest and prompt further exploration instead of making people uncertain or overloaded. In our view, this does not necessarily mean that flooding will be unsuccessful. Only that more effort (more content, more sources, more exposure) is required to make audiences feel that the ‘debate’ – while interesting at first – is slowly becoming difficult to follow and unworthy of cognitive investment.

Provided that enough ‘erosion’ has taken place, our model predicts that audiences eventually abandon the flooded topic, becoming unable and unwilling to reach a clear verdict which in turn could serve as a basis for action. The clearest real‐world case of this is voter suppression. Here, political communicators no longer try to win opposition voters over to their cause, but attempt to confuse them so that they stay home and do not vote for anyone at all (Wilder, 2021). We also find it imaginable that repeated flooding attempts taking place inside one communication environment – say, a social media site – should have an effect on the perception of this environment. Badly maintained networks that are repeatedly flooded can even produce a cynical impression that truth cannot be found at all – as all that exists are opinions. Again, the one‐shot experimental designs in this paper were not designed to pick up on these effects. To this end, more ecologically valid simulated environments are needed. We believe this is a fruitful avenue for research. If it is consistently shown that such strategies damage the ergonomics of social media sites and potentially lower engagement metrics, then that is a strong argument for parent companies to enact policies against disinformation.

What policies might these be? The flooding model provides us with some guidance on which interventions are likely to work – and which ones may backfire. For example, fact‐checking each inaccurate message circulated in a flooding operation seems poorly suited to address the problem: it keeps adding more incongruent content to an already saturated communication environment, where the real danger is not that audiences will believe the falsehoods, but that they will not find reliable information. One might say that increasing the ratio of accurate information compared to inaccurate ones should have an effect, even within our model. We believe, however, that this runs into what is known as the ‘scalability problem’ of disinformation (Celadin et al., 2023): low‐quality, flooding messages are less costly to create (as they are not bound by truth and editorial rules), while generating fact‐checks remains a costly, time‐consuming activity, meaning that trying to match the flooding strategy by quantity would quickly put reliable information on the back foot. A better strategy would be to amplify reliable signals (Acerbi et al., 2022) through increasing the visibility of reliable sources with a good track record. This, in theory, should lower the cognitive costs of finding and processing accurate information. Strengthening existing institutions that do a good enough job in curating information (Szegőfi & Heintz, 2022) is the longer‐term version of the same logic.

## AUTHOR CONTRIBUTIONS

**Ákos Szegőfi:** Conceptualization; investigation; writing – original draft; visualization; formal analysis; data curation. **Sacha Altay:** Conceptualization; writing – review and editing; data curation; methodology. **Christophe Heintz:** Conceptualization; writing – review and editing; supervision; funding acquisition.

## CONFLICT OF INTEREST STATEMENT

The authors declare no conflicts of interest.

## ETHICAL APPROVAL

This research has been approved by the Austrian Psychological Research Ethics Board (PREBO). Ref. no.: 2023‐30.

## Supporting information


Tables A1–A19

## Data Availability

The data that support the findings of this study are openly available in OSF, under the DOI 10.17605/OSF.IO/F58TN (anonymized link provided in the manuscript). This study has been pre‐registered prior to data collection. The anonymized project and the data are available under the following anonymous https://osf.io/f58tn/overview?view_only=00ddad273592466d8be2709b6dc2f341. Anonymized pre‐registration is available under the following https://osf.io/aebxz/overview?view_only=db192803c86e4043911ad733bb7f4de1.

## Footnotes

^1^
We acknowledge that other, potentially meaningful comparisons are possible that vary the ratios of accurate and inaccurate statements. The contrast used here was selected for the reason that it involved two very different environments, so that it could serve as a benchmark for more specialized studies. However, if no effect shows even with such a basic contrast, then more specialised studies (with much larger samples due to the multiplication of conditions) could be spared. We return to the consequences of this limitation in the discussion section.

^2^
We would like to thank our reviewers for pushing us to think about and address this question.
