# Supplementary material for: ‘Flooding’ using disinformation
Source: Br J Soc Psychol. 2026 Nov 8;66(1):e70151. doi: 10.1111/bjso.70151 (PMC13745977; doi:10.1111/bjso.70151)
Supplement: Supplementary file 1 [file BJSO-66-0-s001.docx]

Supplementary materials

Study 1: Sunscreen experiment descriptives, regression tables, and model specifications

*Table A1. Descriptives of Study 1 (sunscreens)*

| **Variable** | ***M*** | ***SD*** | **Median** | **Min** | **Max** | **Skew** | **Kurtosis** | ***n*** |
| --- | --- | --- | --- | --- | --- | --- | --- | --- |
| Age | 41.87 | 12.91 | 40.00 | 18 | 95 | 0.55 | –0.07 | 1307 |
| Sex (1 = Female) | 1.45 | 0.50 | 1.00 | 1 | 3 | 0.24 | –1.85 | 1307 |
| Condition (1/2) | 1.50 | 0.50 | 2.00 | 1 | 2 | 0.00 | –2.00 | 1307 |
| Pre-treatment attitude | 5.76 | 1.28 | 6.00 | 1 | 7 | –1.39 | 2.13 | 1307 |
| Post-treatment attitude | 6.10 | 1.15 | 6.00 | 1 | 7 | –1.90 | 4.38 | 1307 |
| Accuracy (accurate items) | 5.12 | 1.64 | 6.00 | 1 | 7 | –0.87 | 0.00 | 1307 |
| Accuracy (inaccurate items) | 3.82 | 1.56 | 4.00 | 1 | 7 | –0.10 | –0.63 | 1307 |
| Information overload | 4.02 | 1.75 | 5.00 | 1 | 7 | –0.16 | –1.08 | 1307 |

*Table A2. Correlations among Sunscreen-study variables*

| **Variable_1** | **Variable_2** | **r** | **95% CI** | **p** | **N** |
| --- | --- | --- | --- | --- | --- |
| Age | sex_num | 0.04 | [-0.02, 0.09] | 0.203 | 1,305 |
| Age | attitude_pre | -0.06 | [-0.12, -0.01] | 0.022 | 1,307 |
| Age | attitude_post | -0.06 | [-0.12, -0.01] | 0.023 | 1,307 |
| Age | accurate | -0.10 | [-0.15, -0.05] | <.001 | 1,307 |
| Age | inaccurate | -0.02 | [-0.08, 0.03] | 0.417 | 1,307 |
| Age | overload | 0.00 | [-0.05, 0.05] | 0.980 | 1,307 |
| Age | cond_num | -0.02 | [-0.08, 0.03] | 0.395 | 1,307 |
| sex_num | attitude_pre | -0.04 | [-0.1, 0.01] | 0.121 | 1,305 |
| sex_num | attitude_post | -0.02 | [-0.07, 0.04] | 0.512 | 1,305 |
| sex_num | accurate | -0.04 | [-0.1, 0.01] | 0.113 | 1,305 |
| sex_num | inaccurate | 0.09 | [0.04, 0.14] | 0.001 | 1,305 |
| sex_num | overload | 0.12 | [0.06, 0.17] | <.001 | 1,305 |
| sex_num | cond_num | 0.02 | [-0.03, 0.07] | 0.454 | 1,305 |
| attitude_pre | attitude_post | 0.75 | [0.73, 0.77] | <.001 | 1,307 |
| attitude_pre | accurate | 0.44 | [0.39, 0.48] | <.001 | 1,307 |
| attitude_pre | inaccurate | -0.38 | [-0.43, -0.33] | <.001 | 1,307 |
| attitude_pre | overload | -0.27 | [-0.32, -0.22] | <.001 | 1,307 |
| attitude_pre | cond_num | -0.07 | [-0.13, -0.02] | 0.010 | 1,307 |
| attitude_post | accurate | 0.41 | [0.36, 0.45] | <.001 | 1,307 |
| attitude_post | inaccurate | -0.32 | [-0.37, -0.27] | <.001 | 1,307 |
| attitude_post | overload | -0.15 | [-0.21, -0.1] | <.001 | 1,307 |
| attitude_post | cond_num | 0.01 | [-0.05, 0.06] | 0.813 | 1,307 |
| accurate | inaccurate | -0.16 | [-0.22, -0.11] | <.001 | 1,307 |
| accurate | overload | -0.21 | [-0.26, -0.16] | <.001 | 1,307 |
| accurate | cond_num | -0.06 | [-0.12, -0.01] | 0.020 | 1,307 |
| inaccurate | overload | 0.30 | [0.25, 0.35] | <.001 | 1,307 |
| inaccurate | cond_num | 0.08 | [0.02, 0.13] | 0.006 | 1,307 |
| overload | cond_num | 0.15 | [0.1, 0.2] | <.001 | 1,307 |

*Note.* Values are Pearson correlations. 95% confidence intervals were computed using Fisher’s z transformation. Sex and condition were coded as binary (0/1); denoted as sex_num and cond_num, respectively. Responses of “prefer not to say” were treated as missing (N = 2 observations in the sample). Pairwise Ns are reported due to missingness.

*Table A3. Linear regression prediction post-treatment Sunscreen attitude*

| **Predictor** | **b** | **SE** | ***t*** | **95% CI** | ***p*** |
| --- | --- | --- | --- | --- | --- |
| (Intercept) | 2.177 | 0.127 | – | [1.928, 2.426] | – |
| Pre-treatment attitude | 0.680 | 0.017 | 41.207 | [0.648, 0.713] | < .001 |
| Condition (Treatment) | 0.138 | 0.042 | 3.281 | [0.056, 0.221] | .001 |
| Age | -0.001 | 0.002 | -0.794 | [-0.005, 0.002] | .428 |
| Sex (Male) | -0.031 | 0.042 | -0.737 | [-0.114, 0.052] | .461 |
| Sex (Prefer not to say) | 1.070 | 0.538 | 1.988 | [0.014, 2.126] | .047 |

*Note.* Model statistics: N = 1307; R² = .568; Adjusted R² = .567; AIC = 3000.1; BIC = 3036.4; RMSE = 0.76; df = 1301

*Table A4. Linear regression predicting the accuracy ratings of accurate Sunscreen posts*

| **Predictor** | **b** | **SE** | ***t*** | **95% CI** | ***p*** |
| --- | --- | --- | --- | --- | --- |
| (Intercept) | 5.696 | 0.167 | – | [5.369, 6.024] | – |
| Condition (Treatment) | -0.216 | 0.090 | -2.404 | [-0.393, -0.040] | .016 |
| Age | -0.013 | 0.003 | -3.615 | [-0.019, -0.006] | < .001 |
| Sex (Male) | 0.129 | 0.091 | 1.419 | [-0.049, 0.306] | .156 |
| Sex (Prefer not to say) | 0.480 | 1.152 | 0.417 | [-1.780, 2.739] | .677 |

*Note.* Model statistics: N = 1307; R² = .016; Adjusted R² = .013; AIC = 4987.5; BIC = 5018.5; RMSE = 1.62; df = 1302

*Table A5. Linear regression predicting the accuracy ratings of inaccurate Sunscreen posts*

| **Predictor** | **b** | **SE** | ***t*** | **95% CI** | ***p*** |
| --- | --- | --- | --- | --- | --- |
| (Intercept) | 3.949 | 0.159 | – | [3.638, 4.260] | – |
| Condition (Treatment) | 0.228 | 0.086 | 2.670 | [0.061, 0.396] | .008 |
| Age | -0.003 | 0.003 | -0.881 | [-0.009, 0.004] | .378 |
| Sex (Male) | -0.278 | 0.086 | -3.223 | [-0.447, -0.109] | .001 |
| Sex (Prefer not to say) | 1.568 | 1.095 | 1.433 | [-0.579, 3.716] | .152 |

*Note.* Model statistics: N = 1307; R² = .016; Adjusted R² = .013; AIC = 4854.7; BIC = 4885.7; RMSE = 1.54; df = 1302

*Table A6. Linear regression predicting perceived information overload*

| **Predictor** | **b** | **SE** | ***t*** | **95% CI** | ***p*** |
| --- | --- | --- | --- | --- | --- |
| (Intercept) | 3.932 | 0.176 | – | [3.587, 4.278] | – |
| Condition (Treatment) | 0.515 | 0.095 | 5.427 | [0.329, 0.701] | < .001 |
| Age | -0.000 | 0.004 | -0.016 | [-0.007, 0.007] | .987 |
| Sex (Male) | -0.396 | 0.096 | -4.146 | [-0.584, -0.209] | < .001 |
| Sex (Prefer not to say) | 2.813 | 1.214 | 2.316 | [0.430, 5.195] | .021 |

*Note.* Model statistics: N = 1307; R² = .040; Adjusted R² = .037; AIC = 5126.2; BIC = 5157.3; RMSE = 1.71; df = 1302

*Table A8. Exploratory analysis on truth-discernment using linear mixed effects model.*

| **Predictor** | | **b** | **SE** | **95% CI** | | **df** | **t** | **p** |
| --- | --- | --- | --- | --- | --- | --- | --- | --- |
| Intercept (Inaccurate–Control) | | 3.70 | 0.07 | [3.57, 3.83] | | – | – | – |
| Discernment (Accurate vs. Inaccurate) | | 1.52 | 0.09 | [1.35, 1.69] | | 2525.85 | 17.40 | < .001 |
| Condition (Treatment vs. Control) | | 0.24 | 0.10 | [0.04, 0.43] | | 182.89 | 2.38 | .018 |
| Discernment × Condition | | –0.45 | 0.12 | [-0.69, -0.20] | | 2525.85 | –3.62 | < .001 |
| **Random Effect** | **Variance** | **SD** |  | |  |  |  |  |
| Participant (Intercept) | 0.000 | 0.000 |  | |  |  |  |  |
| Item (Intercept) | 0.038 | 0.196 |  | |  |  |  |  |
| Residual | 2.498 | 1.580 |  | |  |  |  |  |

*Note.* Reference categories are *inaccurate* (for Discernment) and *control* (for Condition).
Model statistics: N = 1307; AIC = 9856.6; BIC = 9897.7; logLik = –4921.3; deviance = 9842.6.

Study 2: GMO experiment descriptives, regression tables, and model specifications

*Table A9. Descriptives of Study 2 (GMOs)*

| **Variable** | ***M*** | ***SD*** | **Median** | **Min** | **Max** | **Skew** | **Kurtosis** | ***n*** |
| --- | --- | --- | --- | --- | --- | --- | --- | --- |
| Age | 41.51 | 12.74 | 40.00 | 18 | 82 | 0.51 | –0.36 | 1300 |
| Sex (1 = Female) | 1.45 | 0.50 | 1.00 | 1 | 3 | 0.25 | –1.84 | 1300 |
| Condition (1/2) | 1.50 | 0.50 | 2.00 | 1 | 2 | –0.01 | –2.00 | 1300 |
| Pre-treatment attitude 1 | 4.33 | 1.69 | 5.00 | 1 | 7 | –0.34 | –0.74 | 1300 |
| Post-treatment attitude 1 | 4.21 | 1.71 | 4.00 | 1 | 7 | –0.25 | –0.84 | 1300 |
| Pre-treatment attitude 2 | 4.11 | 1.57 | 4.00 | 1 | 7 | –0.21 | –0.57 | 1300 |
| Post-treatment attitude 2 | 4.00 | 1.58 | 4.00 | 1 | 7 | –0.09 | –0.63 | 1300 |
| Accuracy (accurate items) | 4.63 | 1.47 | 5.00 | 1 | 7 | –0.57 | –0.12 | 1300 |
| Accuracy (inaccurate items) | 4.15 | 1.69 | 4.00 | 1 | 7 | –0.20 | –0.84 | 1300 |
| Information overload | 4.77 | 1.64 | 5.00 | 1 | 7 | –0.66 | –0.45 | 1300 |

*Table A10. Correlations among GMO-study variables.*

| **Variable_1** | **Variable_2** | **r** | **95% CI** | **p** | **N** |
| --- | --- | --- | --- | --- | --- |
| Age | sex_num | 0.05 | [0, 0.11] | 0.060 | 1,298 |
| Age | attitude_pre1 | -0.08 | [-0.13, -0.02] | 0.004 | 1,300 |
| Age | attitude_post1 | -0.08 | [-0.14, -0.03] | 0.002 | 1,300 |
| Age | attitude_pre2 | -0.05 | [-0.1, 0.01] | 0.103 | 1,300 |
| Age | attitude_post2 | -0.05 | [-0.1, 0.01] | 0.078 | 1,300 |
| Age | accurate | -0.07 | [-0.12, -0.01] | 0.019 | 1,300 |
| Age | inaccurate | 0.01 | [-0.04, 0.07] | 0.680 | 1,300 |
| Age | overload | 0.04 | [-0.01, 0.09] | 0.144 | 1,300 |
| Age | cond_num | 0.00 | [-0.05, 0.05] | 0.998 | 1,300 |
| sex_num | attitude_pre1 | -0.20 | [-0.25, -0.15] | <.001 | 1,298 |
| sex_num | attitude_post1 | -0.22 | [-0.27, -0.17] | <.001 | 1,298 |
| sex_num | attitude_pre2 | -0.21 | [-0.26, -0.16] | <.001 | 1,298 |
| sex_num | attitude_post2 | -0.20 | [-0.26, -0.15] | <.001 | 1,298 |
| sex_num | accurate | -0.15 | [-0.2, -0.1] | <.001 | 1,298 |
| sex_num | inaccurate | 0.14 | [0.08, 0.19] | <.001 | 1,298 |
| sex_num | overload | 0.15 | [0.1, 0.2] | <.001 | 1,298 |
| sex_num | cond_num | 0.02 | [-0.03, 0.08] | 0.446 | 1,298 |
| attitude_pre1 | attitude_post1 | 0.89 | [0.88, 0.9] | <.001 | 1,300 |
| attitude_pre1 | attitude_pre2 | 0.82 | [0.8, 0.84] | <.001 | 1,300 |
| attitude_pre1 | attitude_post2 | 0.75 | [0.72, 0.77] | <.001 | 1,300 |
| attitude_pre1 | accurate | 0.43 | [0.39, 0.47] | <.001 | 1,300 |
| attitude_pre1 | inaccurate | -0.48 | [-0.52, -0.44] | <.001 | 1,300 |
| attitude_pre1 | overload | -0.22 | [-0.27, -0.16] | <.001 | 1,300 |
| attitude_pre1 | cond_num | 0.02 | [-0.03, 0.08] | 0.434 | 1,300 |
| attitude_post1 | attitude_pre2 | 0.76 | [0.74, 0.78] | <.001 | 1,300 |
| attitude_post1 | attitude_post2 | 0.82 | [0.8, 0.83] | <.001 | 1,300 |
| attitude_post1 | accurate | 0.45 | [0.41, 0.5] | <.001 | 1,300 |
| attitude_post1 | inaccurate | -0.51 | [-0.55, -0.47] | <.001 | 1,300 |
| attitude_post1 | overload | -0.24 | [-0.29, -0.19] | <.001 | 1,300 |
| attitude_post1 | cond_num | -0.03 | [-0.08, 0.02] | 0.271 | 1,300 |
| attitude_pre2 | attitude_post2 | 0.86 | [0.85, 0.87] | <.001 | 1,300 |
| attitude_pre2 | accurate | 0.39 | [0.35, 0.44] | <.001 | 1,300 |
| attitude_pre2 | inaccurate | -0.42 | [-0.47, -0.38] | <.001 | 1,300 |
| attitude_pre2 | overload | -0.20 | [-0.25, -0.15] | <.001 | 1,300 |
| attitude_pre2 | cond_num | 0.04 | [-0.02, 0.09] | 0.176 | 1,300 |
| attitude_post2 | accurate | 0.40 | [0.36, 0.45] | <.001 | 1,300 |
| attitude_post2 | inaccurate | -0.47 | [-0.51, -0.43] | <.001 | 1,300 |
| attitude_post2 | overload | -0.22 | [-0.27, -0.16] | <.001 | 1,300 |
| attitude_post2 | cond_num | -0.03 | [-0.08, 0.03] | 0.292 | 1,300 |
| accurate | inaccurate | -0.20 | [-0.25, -0.15] | <.001 | 1,300 |
| accurate | overload | -0.09 | [-0.14, -0.04] | 0.001 | 1,300 |
| accurate | cond_num | -0.05 | [-0.11, 0] | 0.049 | 1,300 |
| inaccurate | overload | 0.28 | [0.23, 0.33] | <.001 | 1,300 |
| inaccurate | cond_num | 0.04 | [-0.02, 0.09] | 0.187 | 1,300 |
| overload | cond_num | 0.07 | [0.01, 0.12] | 0.014 | 1,300 |

*Note.* Values are Pearson correlations. 95% confidence intervals were computed using Fisher’s z transformation. Sex and condition were coded as binary (0/1), denoted as sex_num and cond_num in the table, respectively. Responses of “prefer not to say” were treated as missing (N = 2 observations in the sample). Pairwise Ns are reported due to missingness.

*Table A11. Linear regression predicting post-exposure GMO Attitude 1*

| **Predictor** | **b** | **SE** | ***t*** | **95% CI** | ***p*** |
| --- | --- | --- | --- | --- | --- |
| (Intercept) | 0.424 | 0.097 | – | [0.233, 0.615] | – |
| Pre-treatment attitude | 0.895 | 0.013 | 69.060 | [0.869, 0.920] | < .001 |
| Condition (Treatment) | –0.167 | 0.043 | –3.899 | [–0.250, –0.083] | < .001 |
| Age | –0.002 | 0.002 | –0.994 | [–0.005, 0.002] | .321 |
| Sex (Male) | 0.139 | 0.044 | 3.163 | [0.053, 0.225] | .002 |
| Sex (Prefer not to say) | 0.216 | 0.545 | 0.396 | [–0.854, 1.286] | .692 |

*Note.* Model statistics: N = 1300; R² = .798; Adjusted R² = .798; AIC = 3014.5; BIC = 3050.7; RMSE = 0.77, df = 1294

*Table A12. Linear regression predicting post-exposure GMO Attitude 2*

| **Predictor** | **b** | **SE** | ***t*** | **95% CI** | ***p*** |
| --- | --- | --- | --- | --- | --- |
| (Intercept) | 0.567 | 0.100 | – | [0.371, 0.764] | – |
| Pre-treatment attitude | 0.864 | 0.015 | 59.462 | [0.835, 0.892] | < .001 |
| Condition (Treatment) | –0.192 | 0.044 | –4.326 | [–0.280, –0.105] | < .001 |
| Age | –0.001 | 0.002 | –0.639 | [–0.005, 0.002] | .523 |
| Sex (Male) | 0.060 | 0.046 | 1.308 | [–0.030, 0.150] | .191 |
| Sex (Prefer not to say) | 0.154 | 0.568 | 0.271 | [–0.959, 1.267] | .786 |

*Note.* Model statistics: N = 1300; R² = .744; Adjusted R² = .743; AIC = 3118.1; BIC = 3154.3; RMSE = 0.80, df = 1294

*Table A13. Linear regression predicting accuracy ratings of accurate GMO posts*

| **Predictor** | **b** | **SE** | ***t*** | **95% CI** | ***p*** |
| --- | --- | --- | --- | --- | --- |
| (Intercept) | 4.791 | 0.150 | – | [4.496, 5.085] | – |
| Condition (Treatment) | –0.151 | 0.081 | –1.867 | [–0.309, 0.008] | .062 |
| Age | –0.007 | 0.003 | –2.098 | [–0.013, –0.000] | .036 |
| Sex (Male) | 0.432 | 0.081 | 5.305 | [0.272, 0.592] | < .001 |
| Sex (Prefer not to say) | 0.453 | 1.032 | 0.439 | [–1.572, 2.477] | .661 |

*Note.* Model statistics: N = 1300; R² = .028; Adjusted R² = .025; AIC = 4672.7; BIC = 4703.7; RMSE = 1.45 df = 1295

*Table A14. Linear regression predicting accuracy ratings of inaccurate GMO posts*

| **Predictor** | **b** | **SE** | ***t*** | **95% CI** | ***p*** |
| --- | --- | --- | --- | --- | --- |
| (Intercept) | 4.277 | 0.173 | – | [3.938, 4.616] | – |
| Condition (Treatment) | 0.112 | 0.093 | 1.206 | [–0.070, 0.295] | .228 |
| Age | 0.001 | 0.004 | 0.150 | [–0.007, 0.008] | .881 |
| Sex (Male) | –0.462 | 0.094 | –4.932 | [–0.646, –0.278] | < .001 |
| Sex (Prefer not to say) | –0.797 | 1.187 | –0.672 | [–3.126, 1.532] | .502 |

*Note.* Model statistics: N = 1300; R² = .020; Adjusted R² = .017; AIC = 5037.4; BIC = 5068.4; RMSE = 1.67 df = 1295

*Table A15. Linear regression predicting perceived information overload*

| **Predictor** | **b** | **SE** | ***t*** | **95% CI** | ***p*** |
| --- | --- | --- | --- | --- | --- |
| (Intercept) | 4.697 | 0.167 | – | [4.369, 5.026] | – |
| Condition (Treatment) | 0.214 | 0.090 | 2.374 | [0.037, 0.391] | .018 |
| Age | 0.004 | 0.004 | 1.202 | [–0.003, 0.011] | .230 |
| Sex (Male) | –0.485 | 0.091 | –5.347 | [–0.663, –0.307] | < .001 |
| Sex (Prefer not to say) | 0.147 | 1.150 | 0.128 | [–2.109, 2.403] | .898 |

*Note.* Model statistics: N = 1300; R² = .028; Adjusted R² = .025; AIC = 4954.7; BIC = 4985.7; RMSE = 1.62; df = 1295

*Table A16. Linear regression predicting accuracy ratings of accurate posts based on GMO Attitude 1*

| **Predictor** | **b** | **SE** | ***t*** | **95% CI** | ***p*** |
| --- | --- | --- | --- | --- | --- |
| (Intercept) | 3.199 | 0.168 | – | [2.870, 3.528] | – |
| Pre-treatment attitude | 0.365 | 0.022 | – | [0.321, 0.409] | – |
| Condition (Treatment) | –0.184 | 0.074 | –2.501 | [–0.329, –0.040] | .012 |
| Age | –0.003 | 0.003 | –1.159 | [–0.009, 0.002] | .247 |
| Sex (Male) | 0.184 | 0.076 | 2.433 | [0.036, 0.333] | .015 |
| Sex (Prefer not to say) | –0.084 | 0.940 | –0.090 | [–1.929, 1.760] | .928 |

*Note.* Model statistics: N = 1300; R² = .195; Adjusted R² = .192; AIC = 4430.6; BIC = 4466.8; RMSE = 1.32; df = 1294

*Table A17. Linear regression predicting accuracy ratings of inaccurate posts based on GMO Attitude 1*

| **Predictor** | **b** | **SE** | ***t*** | **95% CI** | ***p*** |
| --- | --- | --- | --- | --- | --- |
| (Intercept) | 6.355 | 0.187 | – | [5.988, 6.723] | – |
| Pre-treatment attitude | –0.477 | 0.025 | –19.128 | [–0.526, –0.428] | < .001 |
| Condition (Treatment) | 0.156 | 0.082 | 1.894 | [–0.006, 0.317] | .058 |
| Age | –0.004 | 0.003 | –1.163 | [–0.010, 0.003] | .245 |
| Sex (Male) | –0.139 | 0.084 | –1.639 | [–0.304, 0.027] | .101 |
| Sex (Prefer not to say) | –0.096 | 1.049 | –0.092 | [–2.155, 1.962] | .927 |

*Note.* Model statistics: N = 1300; R² = .236; Adjusted R² = .233; AIC = 4715.7; BIC = 4751.9; RMSE = 1.48; df = 1294

*Table A18. Linear regression predicting accuracy ratings of accurate posts based on GMO Attitude 2*

| **Predictor** | **b** | **SE** | ***t*** | **95% CI** | ***p*** |
| --- | --- | --- | --- | --- | --- |
| (Intercept) | 3.387 | 0.169 | – | [3.055, 3.719] | – |
| Pre-treatment attitude | 0.358 | 0.024 | 14.621 | [0.310, 0.406] | < .001 |
| Condition (Treatment) | –0.199 | 0.075 | –2.656 | [–0.346, –0.052] | .008 |
| Age | –0.005 | 0.003 | –1.756 | [–0.011, 0.001] | .079 |
| Sex (Male) | 0.192 | 0.077 | 2.488 | [0.041, 0.344] | .013 |
| Sex (Prefer not to say) | 0.011 | 0.957 | 0.012 | [–1.866, 1.888] | .991 |

*Note.* Model statistics: N = 1300; R² = .166; Adjusted R² = .163; AIC = 4475.9; BIC = 4512.1; RMSE = 1.35; df = 1294

*Table A19. Linear regression predicting accuracy ratings of inaccurate posts based on GMO Attitude 2*

| **Predictor** | **b** | **SE** | ***t*** | **95% CI** | ***p*** |
| --- | --- | --- | --- | --- | --- |
| (Intercept) | 6.033 | 0.192 | – | [5.657, 6.409] | – |
| Pre-treatment attitude | –0.448 | 0.028 | –16.137 | [–0.502, –0.393] | < .001 |
| Condition (Treatment) | 0.172 | 0.085 | 2.030 | [0.006, 0.339] | .043 |
| Age | –0.001 | 0.003 | –0.394 | [–0.008, 0.005] | .693 |
| Sex (Male) | –0.162 | 0.088 | –1.853 | [–0.334, 0.010] | .064 |
| Sex (Prefer not to say) | –0.245 | 1.084 | –0.226 | [–2.372, 1.882] | .821 |

*Note.* Model statistics: N = 1300; R² = .184; Adjusted R² = .181; AIC = 4801.0; BIC = 4837.2; RMSE = 1.53, df = 1294
